# The Role of Pain Acceptance, Pain Catastrophizing, and Coping Strategies: A Validation of the Common Sense Model in Females Living with Fibromyalgia

**DOI:** 10.1007/s10880-022-09873-w

**Published:** 2022-05-03

**Authors:** Kathryn M. Droppert, Simon Robert Knowles

**Affiliations:** 1 Faculty of Health, Arts and Design, Swinburne University of Technology, Hawthorn, PO Box 218, Melbourne, 3122 Australia; 2 Faculty of Medicine, Dentistry, & Health Sciences, The University of Melbourne, Melbourne, Australia; 3 Colorectal Medicine and Genetics, The Royal Melbourne Hospital, Melbourne, Australia

**Keywords:** Fibromyalgia, Common sense model, Psychological distress, Quality of life

## Abstract

This study aimed to examine the extent to which illness beliefs, coping styles, pain acceptance, pain catastrophizing, and psychological distress mediate the relationship between fibromyalgia symptoms and quality of life (QoL) in a female cohort diagnosed with Fibromyalgia (*n* = 151). Measures used included the Revised Fibromyalgia Impact Questionnaire, Carver Brief COPE scale, Chronic Pain Acceptance Questionnaire Revised, Pain Catastrophizing Scale, Brief Illness Perceptions Questionnaire, Depression and Anxiety Stress Scales, and European Health Interview Survey Quality of Life 8-item Index. Using structural equation modelling, the final model indicated that fibromyalgia symptom severity had a significant direct influence on illness perceptions and psychological distress. In turn, illness perceptions had a significant direct influence on maladaptive coping, pain catastrophizing, pain acceptance, and QoL. Pain catastrophizing and maladaptive coping influenced psychological distress, and in turn distress impacted QoL. Acceptance of pain was found to be influenced by maladaptive coping and in turn acceptance of pain influenced QoL.

Fibromyalgia (FM) is a painful medical condition associated with poor treatment outcomes and quality of life (QoL) (Arnold et al., 2011; Choy et al., 2010; Clark et al., 2013; Skaer & Kwong, 2017). Compared to other pain-related illnesses, autoimmune disorders, and the general public, individuals with FM report higher psychological distress (Capraro et al., 2012; Lami et al., 2018; McInnis et al., 2014; Schaefer et al., 2016; Toussaint et al., 2019). Additionally, higher FM symptoms have been shown to relate to greater psychological distress (PD) and poorer QoL (De Souza Santos Berber et al., 2005; Lee et al., 2017; Schaefer et al., 2011; Toussaint et al., 2017).

FM-cohort studies have demonstrated that illness beliefs and coping strategies can significant impact psychological distress, functional impairment, and pain intensity (Alok et al., 2014; Boehm et al., 2011; Cui et al., 2009; Smith et al., 2009; Stuifbergen et al., 2006).

Given the significant individual and societal burdens associated with FM and the limited efficacy of current standard diagnosis and treatment (Chandran et al., 2012; Choy et al., 2010; Clark et al., 2013; Guymer et al., 2016; Skaer & Kwong, 2017; Vervoort et al., 2016; Walitt et al., 2015), further research is needed to understand the degree to which modifiable psychosocial mediators of adaption (such as acceptance, coping strategies, and pain catastrophizing) influence physical and mental health and QoL outcomes.

The Common Sense Model (CSM; Leventhal et al., 1980; See Fig. 1) is a well-established and valuable theoretical model that posits illness severity influences illness perceptions (e.g. beliefs about the cause, consequences). Additionally, illness perceptions act as a mediator between illness severity and illness outcomes (e.g. psychological distress and QoL) and influence the coping strategies utilized by individuals to manage the impact of their illness and perceptions on illness outcomes (Hagger & Orbell, 2003; Hagger et al., 2017). To date, substantial evidence supports the model across multiple chronic illness conditions (see Hagger & Orbell, 2003, for review); however, no research has utilized the CSM in a FM cohort.

**Fig. 1:**
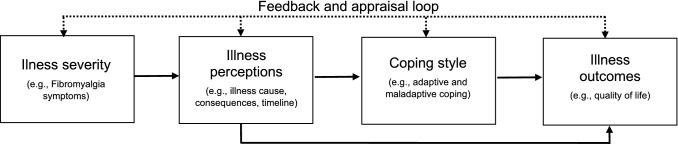
The common sense model (Hagger & Orbell, 2003), adapted by Knowles et al. (2011)

Other psychological variables identified in the FM literature which may also be useful to extend the CSM include pain acceptance and pain catastrophizing. Research has demonstrated that greater pain acceptance (the willingness to live with pain without trying to change, avoid, or reduce it, McCracken, 1999) is associated with reduced psychological distress, while pain catastrophizing (the exaggerated and negative appraisal or actual or anticipated painful experiences) is associated with increased distress and pain-related disability, and poorer QoL in FM populations (Burckhardt et al., 1997; Edwards et al., 2006; Lami et al., 2018; Rodero et al., 2011).

The current literature suggests that despite strong evidence of associations between illness severity, illness perceptions, pain acceptance, pain catastrophizing, coping styles, psychological distress, and QoL in FM, no study to the authors’ knowledge has evaluated these psychosocial variables together in a FM cohort. Using structural equation modelling (SEM), the aim of the study was to extend the CSM to explore the potential interrelationships between these variables in a FM cohort. It was hypothesized that consistent with the posits of the CSM that illness perceptions, pain acceptance, pain catastrophizing, and coping styles would mediate the relationship between FM symptoms and patient-reported outcomes (psychological distress and QoL).

## Methods

### Participants

A total of 151 participants with a mean age of 45.98 years (SD = 10.57) completed a self-administered online questionnaire. Of the 151 participants, the majority of participants were married (39.07%) or single (23.18%), lived with a partner (60.26%) or alone (29.14%), lived in their own home (44.37%) or in private rental (38.41%), were on a pension (27.15%) or unemployed (37.75%).

On average, participants had experienced symptoms of FM for 11.58 years (SD = 10.75), with 58.90% having experienced fibromyalgia symptoms for more than 5 years. Seventy-seven per cent of participants reported taking medication to help manage the fibromyalgia symptoms. Comorbidity in the participant cohort was also high with 83.44% reported being diagnosed with another physical health condition, with the most common being arthritis (26.49%) and chronic fatigue syndrome (8.61%).

### Measures

*The Revised Fibromyalgia Impact Questionnaire* (*FIQ-R)* (Bennett et al., 2009*)*. The FIQ-R is a 21-item scale (zero to ten response format) used to measure the functional impact, the overall impact of symptoms, and the intensity of symptoms. An overall score (0–100) was obtained by weighting the subscale scores and summing them together with higher scores indicating a greater perceived impact of fibromyalgia symptoms. An error was made when administering the questionnaire, as participants were asked to rate their symptom severity from none to a lot instead of the individualized answers to the questionnaire. Cronbach alpha analysis indicated that the modified scale had strong internal consistency (0.89).

*The Brief Illness Perception Questionnaire (BIPQ)* (Broadbent et al., 2006*)*. The BIPQ is an eight-item scale (zero to ten response format) used to measure cognitive and emotional representations of illness. Consistent with previous research to improve scale consistency Knowles et al. (2011), an exploratory actor analysis using Principal Axis Factoring method with an Oblimin rotation and Cronbach alpha with item-if-deleted analysis recommend the removal of the question “How well do you feel you understand your illness?” (item 7). The remove resulted in a 7-item scale (Cronbach alpha: 0.67) with an overall score obtained by adding all the items together (reversing items three and four). Total scores range from zero to 70 with higher scores indicating a more threatening view of illness.

*The Brief Coping Operations Preference Enquiry *(*Brief COPE*; Carver, 1997*).* The Brief COPE is a 28 item (zero to three response format) that assesses fourteen conceptually different coping response types. Consistent with recommendations by the scale author (Carver, 1997) and previous research (Knowles, et al., 2011), an exploratory factor analysis using the Principal Axis Factoring method with an Oblimin rotation and Cronbach alpha with item-if-deleted analysis was conducted on the scale. Review of the scree plot and pattern matrix suggested a two-factor structure solution. Consistent with previous research (Carver, 1997; Knowles et al., 2011; Knowles, Cook, & Tribbick, 2013), two subscales descriptors (i.e. maladaptive and adaptive coping styles) were used in the study. Maladaptive coping styles refer to items that had an adverse relationship with psychosocial outcomes. Maladaptive coping was composed of 9 items: “I’ve been saying to myself “this isn’t real””, “I’ve been using alcohol or other drugs to make myself feel better”, “I’ve been refusing to believe that it has happened”, “I’ve been saying things to let my unpleasant feeling escape”, “I’ve been using alcohol or other drugs to help me get through it”, “I’ve been criticizing myself”, “I’ve been giving up the attempt to cope”, “I’ve been doing something to think about it less, such as going to the movies, watching TV, reading, daydreaming, sleeping, or shopping”, and “I’ve been blaming myself for things that happened”. Adaptive coping styles refer to items that had a beneficial relationship with psychosocial outcomes and was comprised of 15 items: “I’ve been concentrating my efforts on doing something about the situation I’m in”, “I’ve been getting emotional support from others”, “I’ve been taking action to try and make the situation better”, “I’ve been getting help and advice from other people”, “I’ve been trying to see it in a different light, to make it seem more positive”, “I’ve been trying to come up with a strategy about what to do”, “I’ve been getting comfort and understanding from someone”, “I’ve been looking for something good in what is happening”, “I’ve been making jokes about it”, “I’ve been trying to find comfort in my religion or spiritual beliefs”, “I’ve been trying to get advice or help from other people about what to do”, “I’ve been learning to live with it”, “I’ve been thinking hard about what steps to take”, “I’ve been praying or meditating”, and “I’ve been making fun of the situation”. Factor analysis also suggested the removal of items 1, 6, 20, and 21. Each of the subscale scores is obtained by averaging the items, with higher scores indicating a greater engagement in maladaptive or adaptive coping styles. Maladaptive and adaptive coping were found to have strong internal consistencies (Cronbach alpha 0.75 and 0.86 respectively).

*Chronic Pain Acceptance Questionnaire—Revised (CPAQ-R) *(McCracken et al., 2004). The CPAQ-R is a 20-item scale (zero to six response format) used to measure acceptance of pain. An overall score is obtained by adding the items together. Total scores range from zero to 120 with higher scores indicating greater level of acceptance. Cronbach alpha analysis indicated the modified scale had acceptable internal consistency (0.73).

*Pain Catastrophizing Scale (PCS)* (Sullivan et al., 1995). The PCS is a 13-item scale (zero to four response format) used to measure the degree to which the participant engages in thoughts or feelings that may catastrophize their level of pain. An overall score is obtained by adding the items together. Total scores range from zero to 52 with higher scores indicating greater level of pain catastrophizing. Cronbach alpha analysis indicated that the modified scale had strong internal consistency (0.93).

*Depression Anxiety Stress Scale (DASS) *(Lovibond & Lovibond, 1996)*.* The DASS is a 21-item scale (zero to three response format) used to measure stress, anxiety, and depression levels. An overall score is obtained by adding the items together and multiplying them by two. Total scores range from 0 to 126, with higher scores indicating greater psychological distress. Cronbach alpha analysis indicated that the modified scale had strong internal consistency (0.93).

*The European Health Interview Survey-Quality of Life 8-item Index *(*EUR0HIS-QoL 8-item index*) (Nosikov & Gudex, 2003; Schmidt et al., 2005). The EUROHIS-QoL is an eight-item scale (zero to five response format) used to measure QoL across psychological, physical, social, and environmental domains. An overall score is obtained by adding scores across the items together. Total scores range from eight to 40 with higher scores indicating better QoL. Cronbach alpha analysis indicated the modified scale had strong internal consistency (0.83).

### Procedure

Participants were recruited through FM-related social media (e.g. Australian Fibromyalgia online Facebook forum) and were invited to complete a 20-min online survey. Inclusion criteria were being aged 18 years or older and having a diagnosis of Fibromyalgia. Research was approved by Swinburne University of Technology’s Human Research Ethics Committee (SUHREC). Informed consent was obtained from all research participants. Data were collected from 7 to 9 August 2018.

### Statistical analyses

The data for the present research were analysed using IBM Statistical Package for Social Sciences (SPSS) Version 25. Preliminary data screening was conducted to ensure suitability of the data for further analysis. Analysis of missing values revealed that data were missing completely at random, Little's MCAR test = 7516.83 (8199), *p* > 0.05. There were 26 cases with missing values of over 30%, and these were removed due to the large sample size, consistent with recommendations by Tabachnick and Fiddell (2013), leaving 151 cases for the analysis. Expectation Maximization process was used to replace missing data in the remaining cases (Tabachnick & Fidell, 2013).

Exploratory analysis and visual inspection of the data indicated that all the study variables met the necessary assumptions for statistical analysis (e.g. normality, linearity). Correlational analyses were undertaken to compare the relationship between the study variables. A SEM was specified using the AMOS (Version 27) by an iterative process of adding pathways and removing variables that did not add significantly to the model’s fit. As recommended by Hu and Bentler (1999), pathways were added or removed based on inspection of standardized residuals, AMOS modification indices, the CSM, and prior research on psychosocial factors relating to FM, and a significant improvement in fit (Chi-square goodness of fit test [*χ*^2^] *p* > 0.05; Normed Chi square [*χ*^2^/*N*] = 1–3, Tucker–Lewis index [TLI] > 0.95, Steiger-Lind Root-Mean-Square Error of Approximation [RMSEA] < 0.08, and Standardized Root–Mean-Squared Residual [SRMR] < 0.06).

#### Results

As shown in Table 1 (descriptive and correlational analyses), greater FM symptom severity was associated significantly with poorer illness perceptions and QoL, less pain acceptance, and greater engagement in maladaptive coping, pain catastrophizing, and psychological distress. Poorer illness perceptions were associated significantly with less pain acceptance, greater engagement in maladaptive coping, pain catastrophizing and greater psychological distress, and poorer QoL. Adaptive coping was found only significantly relate to pain acceptance, suggesting that higher engagement in adaptive coping is associated with greater pain acceptance. In contrast, engagement in maladaptive coping was associated significantly with greater pain catastrophizing and psychological distress and poorer QoL. Higher pain acceptance was associated significantly with higher QoL, while engagement in pain catastrophizing was associated significantly with greater psychological distress and poorer QoL.

**Table 1:** Pearson Correlations (and significance values) and descriptive values of CSM variables

	1	2	3	4	5	6	7	Means (SD)
1. FM symptom severity (FIQ-R)	–							67.00 (14.62)
2. Illness perceptions (BIPQ)	.60**	–						54.56 (8.19)
3. Pain acceptance (CPAQ-R)	− .22**	− .30**	–					52.08 (11.95)
4. Pain catastrophizing (PCS)	.45**	.55**	.02	–				26.35 (12.10)
5. Maladaptive coping (COPE)	.32**	.37**	.12	.51**	–			1.83 (.57)
6. Adaptive coping (COPE)	.10	.01	.28**	.02	.09	–		2.29 (.55)
7. Psychological distress (DASS-21)	.62**	.49**	.04	.65**	.64**	.04	–	55.50 (27.13)
8. QoL (EUR0HIS-QOL)	− .50**	− .51**	.44**	− .33**	− .24**	.16	− .44**	18.07 (5.52)

^*^p < .05; ** p < .01

To evaluate the hypothesis, a model of the study variables was conducted. Based on an iterative process guided by Hu and Bentler (1999), adaptive coping and all demographic variables were removed from the model. The final model (see Fig. 1) demonstrated an excellent fit (*χ*^2^ (9) = 15.57, *p* = 0.08, *χ*^2^/*N* = 1.73, CFI = 0.985, TLI = 0.965, RMSEA = 0.070, SRMR = 0.048). The total amount of variance accounted for in each of the variables was also good, 36% of illness perceptions, 14% of maladaptive coping, 15% of pain acceptance, 41% of pain catastrophizing, 64% of psychological distress, and 44% of QoL.

**Fig. 2:**
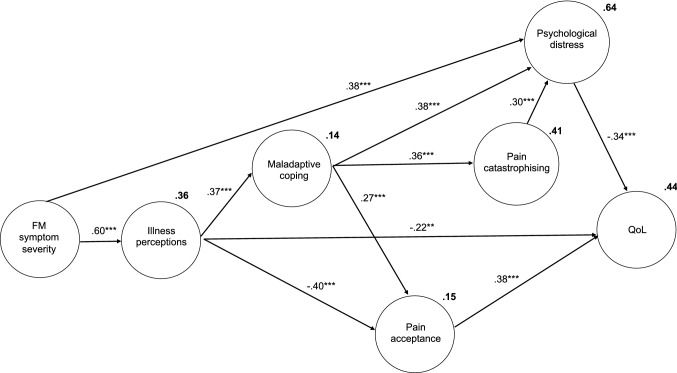
Final extended CSM (note: only latent variables presented with error terms removed; **p < 0.01, ***p < 0.001)

Consistent with the CSM and the study hypotheses, FM symptom severity had a significant direct influence on illness perceptions (*β* = 0.60, *p* < 0.001) and psychological distress (*β* = 0.38, *p* < 0.001). Illness perceptions had a significant direct influence on maladaptive coping (*β* = 0.37, *p* < 0.001), pain catastrophizing (*β* = 0.42, *p* < 0.001), pain acceptance (*β* = − 0.40, *p* < 0.001), and QoL (*β* = − 0.22, *p* < 0.01). Pain catastrophizing and maladaptive coping influenced psychological distress (*β* = 0.30, *p* < 0.001, *β* = 0.38, *p* < 0.001, respectively), and in turn distress impacted QoL (*β* = − 0.34, *p* < 0.001). Acceptance of pain was influenced by maladaptive coping (*β* = 0.27, *p* < 0.001) and in turn acceptance of pain influenced QoL (*β* = 0.38, *p* < 0.001) (Fig. 2).

#### Discussion

Given a high proportion of people diagnosed with FM are women (Yunus et al., 2000), understanding the unique experiences of women who have FM is important to improve health outcomes, as has previously been done in other female-based FM studies (Capraro et al., 2012; McInnis et al., 2014; Stuifbergen et al., 2006). Consistent with past research, this study observed that an increase in FM severity was associated with more negative illness perceptions (Stuifbergen et al., 2006; Van Wilgen et al., 2008), higher pain catastrophizing (Lami et al., 2018), lower pain acceptance (Lami et al., 2018; Rodero et al., 2011), poorer QoL, and higher psychological distress (De Souza Santos Berber et al., 2005; Lee et al., 2017; Schaefer et al., 2011; Toussaint et al., 2017). The results support the study hypothesis that illness perceptions, pain acceptance, pain catastrophizing, and coping styles mediated the relationship between FM symptom severity and patient-reported outcomes (PD and QoL).

The current study is the first to validate the CSM using SEM to simultaneously evaluate the interactions between FM severity, illness perceptions, pain acceptance, pain catastrophizing, coping strategies, psychological distress, and QoL. Consistent with the principles and past research involving the CSM (Hagger & Orbell, 2003; Leventhal et al., 1980), the study found that the severity of FM directly and indirectly influenced psychological distress and QoL. This indirect influence occurred via illness perceptions, and consequent maladaptive coping and pain acceptance. These findings are consistent with previous research demonstrating that illness perceptions act as a mediator between illness severity and QoL (Hagger & Orbell, 2003). The finding is also consistent with FM-based research which demonstrated a relationship between illness severity and illness perceptions (Stuifbergen et al., 2006), and illness severity and QoL (Lee et al., 2017; Schaefer et al., 2011), and illness perceptions and QoL (Capraro et al., 2012; Van Wilgen et al., 2008). Additionally, the study found illness perceptions directly and indirectly influenced QoL via pain catastrophizing, pain acceptance, maladaptive coping, and psychological distress. These findings support the previous research involving FM cohorts which has identified QoL being influenced by pain catastrophizing (Burckhardt et al., 1997; Galvez-Sánchez et al., 2020) and psychological distress (Lee et al., 2017). Prior studies have identified QoL being influenced by pain acceptance in paediatric chronic pain cohorts (McGarrigle et al., 2020) and maladaptive coping in chronic illness cohorts such as diabetes and endometriosis (González-Echevarría et al., 2019; Knowles et al., 2020) but have not specifically been investigated in FM cohorts.

This study highlights the importance of working with individuals living with FM to adapt their perceptions (e.g. how much control and understanding they have of FM, the extent to which their identity is linked to FM diagnosis) of their illness as a way of improving QoL. In addition to addressing comorbid psychological distress, improvement in QoL is likely to be enhanced through reducing pain catastrophizing and promoting strategies to increase pain acceptance. It is noted that while leading pain societies currently recommend the use of cognitive-behavioural therapy in their multicomponent treatment plan for FM (Thieme et al., 2017), the role of increasing pain acceptance has not yet been incorporated into recommendations.

Limitations of this study include self-reported FM diagnosis and severity, and potential for self-selection bias. As data were collected from a FM female-specific online group, results may not reflect the experience of people with more mild symptoms who do not utilize the group. Additionally, as fatigue and cognitive impairment are common components of FM (Arnold et al., 2011), the results may not reflect the experiences of people who experience more severe and debilitating symptoms for whom answering 135 questions may be challenging. The number of participants in this study was below the recommended sample size (> 200) generally recommended when using SEM (Hair et al., 2010); however, the study was based on a validated theoretical model. Due to the administrative error relating to the naming of the anchor points for the FIQ-R, the validity of the scale may have been compromised. Although a factor analysis was completed and the FIQ-R demonstrated a strong internal consistency, further research is needed to replicate the study using the original scale. Further, as the study was cross-sectional, true causal relationships cannot be established. Future research should seek to replicate and extend the current study’s findings within an interventional repeated measures design to explore and evaluate true causality of the study variables and their role in impacting QoL in FM cohorts. Finally, future research should seek to replicate this study and extend it by assessing for potential differences across males and females.

In conclusion, this is the first study to validate the complete CSM in a female cohort with FM using SEM. The findings from this study provides evidence for the interactions between self-reported FM symptom severity, illness perceptions, maladaptive coping, pain acceptance, pain catastrophizing, psychological distress, and QoL. Consistent with the CSM, illness perceptions mediated the relationship between FM severity and QoL, and both pain acceptance and maladaptive coping mediated the relationship between illness perceptions and QoL, and maladaptive coping mediated the relationship between illness perceptions and psychological distress. The study findings suggest that future interventional studies aimed at enhancing QoL in FM cohorts should target psychological variables such as pain acceptance, pain catastrophizing, and maladaptive coping strategies, and illness perceptions.

## Data Availability

The summary data generated during and/or analysed during the current study are available from the corresponding author on reasonable request and after relevant ethical approval.
